# Address

**Published:** 1881-11

**Authors:** 


### THE DENTAL REGISTER.

VOL. XXXV.] NOVEMBER, 1881. [NO. 11.

### ADDRESS.

*Mr. President and Fellow Members of the Massachusetts Dental Society:—*

Never before, oppressed with so many embarrassments, have I presumed to address an assembly like the present. Never before have I been urged, against my own will and better judgment, to stand in the presence of so many of my superiors and elders, to address them upon the vital questions of the hour concerning our chosen profession. I would fain be a silent listener at this time to words of advice and wisdom from the lips of those older in membership in this Society and in our profession, and consequently better fitted to address you than myself. In selecting me for your orator upon this occasion, I feel that you have conferred upon me one of the greatest compliments of my life. For the honor which you thus accord me, accept my sincere thanks.

To-day realizes the successful termination of the sixteenth year of this, the oldest and the representative dental society in Massachusetts. To say that it is an occasion for congratulation, would but feebly express the sentiments of those who have been familiar with the career and watched the growth of our association from its foundation. It is my pleasure to congratulate its founders, many of whom are permitted still to meet with us, in the enjoyment of health and strength, upon the grand triumph achieved, as the result of their labors, in the face of many difficulties. Happily for this yet youthful and aspiring organization, and for those who may hereafter, from this to other generations, be permitted to enjoy its benefits and bear off its honors, the conception, inauguration and prosecution of this work fell into the hands of those whose zeal knew no abatement, and whom no opposition could intimidate or dishearten. Of the forty-two whose names appear upon the Treasurer's books as having been members during the first year of the Society's existence, six have been taken away by death; eleven have either retired from practice or removed from the State, and seventeen still remain members at the present time; thus leaving only eight as the number of those who, still living, and in the practice of dentistry, are not now associated with us. Of the fifteen ex-Presidents of the Society, only two have been removed by death, and all but one of those living still retain their membership in this Society. The positions of honor and trust that have been assigned to those who have been, or are, members of this body, by other dental, medical, and scientific organizations, are many and varied. More than a score of our members have, at various times, been the recipients of the highest honors of other dental societies holding meetings within the limits of the State. One of our number has been called to the highest office in the gift of the American Dental Convention, and two members to the presidency of the American Dental Association. More than thirty of our members have occupied positions of honor and responsibility, either as lecturers, instructors, demonstrators, or professors in dental schools and colleges. These brief statistics show that thus far in our progress as an organization, there has been a good degree of unity among the members, an absence of any disposition to retire from active work in the Society after having obtained its honors; and the fact that we have had as members in this body the leading and active men of our calling in this Commonwealth, and men who are zealous workers for the advancement of our profession. This is a record of which we may well be proud. It is a record of honor to us as an organization, and to us all individually as members; for whatever honors one honors all. It is impossible to estimate, and unnecessary to speak of, the vast amount of good accomplished, through the numberless discussions of professional subjects, and from the many able essays read and clinics held.

That this Society, so auspiciously inaugurated and so prosperously conducted, will continue to meet with the success it has attained during the past, and will gradually extend its influence until the dental profession throughout the State shall recognize the good results attending its establishment, and deem it a high privilege to be enrolled among its members, we sincerely trust and firmly believe. In the long lapse of time, sixteen years is but as a little speck. But in the lives of men and of institutions, a *single year* may be of the very first importance, and give direction and coloring to all the years which are to come. It therefore behooves us to consider well what we, who are of the first generation of membership in this institution, and consequently its first fruits, must do, in order that those who are to follow us will reap the benefit of our wisest counsel and most judicious action; what course we will pursue in the present, so that the future members of this body and of our profession shall be benefitted by our record now. It is pleasant to portray the triumphs of the past, and as a profession we can recite, with commendable pride, the story of the wonderful growth of our calling, from its former obscure position as a mere trade, followed alone by the uneducated and unskilful, to its now acknowledged place among the recognized professions. It is well for us to admire our present condition, and flatter ourselves upon the results accomplished and the work we are now doing. But the great question, the serious business before us, is, are we doing all that can and must be done, in order that the future may be still greater than the present. Every generation is the debtor of future generations. Every act of ours to-day is to exert an inevitable influence over all that are to follow. Every step we take should be so enlightened by experience, so strengthened by exercise, and guarded by caution, as not only to advance us in our professional career, but also prove of inestimable value to those who are to follow us. Ours is one of the youngest, and therefore cannot expect to rank as the first among the professions. But in the sciences there is no first and there is no last. They are all of a common and a noble heritage. Each depends somewhat upon all the others, and draws somewhat from them.

What advances one advances all, since all share in the common development. It does not follow from this, however, that one should not make of his particular art a specialty. Indeed, it is only through devotion to our profession that any of us can hope for success or for any considerable attainment. True it is that in our calling men do not often achieve what are termed the highest prizes of life. Nevertheless, let us "magnify our work," and place our profession, so far as we may, upon a level with those of much older standing. Ours is not one of the showy callings, and affords few occasions for public display; yet, though unpretentious, it presents abundant opportunities for developing the highest qualities of manhood. We do something, at least, for ambition, if our efforts perceptibly advance one of the most humane and beneficient of the arts. We shall deserve to be well spoken of, if we lessen but a grain some of the ills which are a part of our common lot. If faithful to ourselves and to our profession, why should we trouble ourselves about the applause of men? "Nature cares not, although her lovliness should ne'er be seen by human eyes, or praised by human tongues. The cataract exults among the hills, and wears its crown of beauty all alone. Libel the ocean on his tawny sands; write verses in his praise; the unmoved sea erases both alike." We are not commanded to get reputation, to get notoriety; but we are commanded to "get wisdom," to "get understanding, and forsake it not." Let every man wherein he is called, therein abide. That is, let every man be true to his calling, true to his art, however humble. The future reputation of this Society will depend greatly upon the character of its past and present members. It therefore becomes us to place as high as possible the standard of excellence, and we should carefully guard against the admission to our ranks of those who occupy a doubtful or low position in our profession. Our success, as an organization, depends more upon the zeal and earnestness with which we work as co-laborers for the advancement of our calling than upon our numerical strength. I think the time has fully come when we should change our Constitution, so that applicants for admission to our Society shall have first graduated from some respectable medical or dental college, or have received a license from some board of examiners appointed under State law, before they shall be considered eligible to membership in this organization. Other dental associations having a large proportion of their members from among dentists resident in our State, and holding their meeting frequently in our cities and towns, have seen the wisdom of placing this limitation upon their requirements for membership, and have witnessed the good results attending its adoption. Why should we, who are regarded as the representative dental organization in our State, longer make it possible for those who are the veriest charlatans in our midst to be entitled, as far as the qualifications we require are concerned, to come among us, and be of us, simply because they have had a sign denominating them dentists, hung upon some building for the term of five years, and for that length of time have practiced, not in our profession, but upon a too confiding and correspondingly suffering public? It may be true that we have none such now among us, or that it is not often that they seek for admission to this body. But in our present position, we place ourselves upon an equal standing with them. We say to the public that the man who has been in the practice of dentistry for five years, although he may never have gazed upon the pages of a work upon dental science, or may not possess the slightest qualification for such practice in a scientific manner, is just as much entitled to knock at the door of this organization and seek admission to it, as any one, however much he may have studied, or however well qualified he may be, by thorough and special preparation, to enter upon the practice of our calling. Again, our position upon this question is such that we ignore altogether the dental schools and colleges. If a man, without any previous training whatever, can begin, as is not unfrequently done, the practice of dentistry, and after having so practiced for the term of five years, can be entitled to, and gain, admission to this body, simply because of that five years' practice, however bad it may have been, he can say to himself "of what use is it for me to spend my time in attending a dental school? I can be recognized by the representative body of dentists in the State, and the public will then of course recognize me, and I am just as good as any of them." How rediculous would seem the Massachusetts Medical Society, and how little confidence would the great majority of the public have in it, as a body of intelligent and scientific men, if it allowed any person who had simply called himself a physician for five years, the privilege of claiming, for that reason, the right to membership in that body. The question will not bear consideration for a single moment, and yet our position is just as ridiculously absurd as theirs would be if it were such as I have stated. I do not wish to be understood as denying the fact that we have in our profession many men who have never graduated from any medical or dental school, and yet who, by reason of long experience and private study, are well qualified for pursuing their chosen avocation. But I believe the time has fully come when the only legitimate and recognized entrance to our ranks should be through the educational institutions of our specialty, or those of general medicine. These schools are now so numerous, and the facilities for acquiring a proper dental education are so many, that there is no longer any excuse for a person's entering upon the practice of the dental art without first having graduated from some respectable and recognized dental or medical school. I will not argue this question further, although much remains to be said in favor of the adoption of the measure I have urged. I leave the subject in the hope that our Constitution may be speedily changed so that we may occupy a higher and more consistent position.

I do not hesitate to declare, after most mature reflection upon the matter in all its bearings and influences, that it is urgently important that our profession in this State should be also protected, as are the legal and educational professions, by legislative guards and restrictions. Everybody knows that no applicant to the first named of these two is admitted till after he has successfully responded to the severest ordeals of examination by qualified experts. The law accords this justice in order that the members may protect their professions from obtrusive pretenders who have never achieved the intricate knowledge that their theory and practice demand; and the professions accord to the public the protection and defence which such restrictions provide, that the public may not be duped by charlatans, nor misled and harmed by ignorance. By our educational laws, protection is afforded by the restriction that no person shall be employed as a teacher in any school in the State until such person shall have been actually examined, and if found qualified, duly approved by the committee of the city or town wherein such person has applied for appointment. And the law further declares that the Treasurer of such city or town shall refuse payment of the bill of a teacher for service rendered, until he presents a certificate setting forth that examination has been held and approval given. And should any unapproved teacher chastise a pupil for alleged ill-conduct, an action for assault may be maintained. Now with such protection for the law and the school, and with such protection as the clerical profession has provided for itself, all these protections having as their ultimate object, the protection of the public, it would seem that argument was hardly necessary to show its necessity in the profession of the dentist. May we not hope that it will be secured by effort from within our own organization. I know it will be urged that it will be useless to appeal to the legislature, in view of the recent non-success of the physicians and pharmacists in their endeavors to secure such enactments as they have desired. But an examination of the subject will show that the causes which led to their defeat will not apply to us; and I believe that earnest effort upon the part of this Society, through the labors of a committee appointed for the purpose, with such co-operation as may be secured from other societies in the State, will give us a law that will prove of great value to the dental profession and to the public. I most earnestly urge the adoption of such a policy as wise and necessary.

I anticipate neither contradiction nor controversy when I declare my full belief that for the attainment of success and its permanence in every department of human business, higher or lower, education is the indispensible operative power. Education which generates and preserves dignity of soul, purity of character, grace of spirit, power of influence, amplitude of thought, fertility of resource, legitimate self-reliance, assurance of mastery with freedom in action—all these, chastened by thoughtful caution, and beautified by chivalrous devotion to duty. The economic value of each and every man, as an element of society, is increased in the direct ratio of his educational progress, and his ability to advance the general status of his race, in the invention and practical application of new and better methods and means. An educated brain and an educated hand contribute, with most generous certainty, to the happiness and wealth of any country, and this truth having attained foothold in men's minds, the times demand that they who are employed in any occupation in which brain or hand or both are concerned, shall be made more and more expert and better and better able to achieve success. They demand that novices entering upon any profession shall come to the hands of those who are to initiate them therein, less in the raw and more in the ripened condition for recipiency, and so fitted by previous culture that they can be the more easily trained for the new vocation, and be the more surely benefitted by such training. The pig-iron of the furnace must go through much elaboration before it becomes a keen razor or facile scalpel, and the keener it becomes the less pain it inflicts: and the raw boy of the early school must in order not only to attain success, but to satisfy the justifiable demads of the times and of the people, be similarly carried through the necessary processes of further training before he can be ripened into a manhood equal to the exigencies which may press about him. To such education as will so invigorate him as to make him able to master all the possibilities he may meet, he has an innate right; and the consequences that will result to the community withholding or neglecting such training, will in the end redound to its own worst injury. The youth of a nation constitute a *quasi* board of trustees, having in charge the destinies of posterity; and it is eminently true that the youth of America have in charge the destinies of a nation, the future of which, it is safe to prophesy, shadows forward a greatness such as the world has never known; and therefore it is of ineffable importance that the youth of our day and of the coming days be educated up to that degree of power that the complete development of these destinies shall demand. Neither the physical, nor the intellectual, nor the moral condition of either the individual or the community remains at a fixed standpoint. He who does not look upward is in peril of looking downward. He who does not strive to excel, becomes, by slow yet sure degrees, unambitious of excelling; and when excelled by others, he loses ambition and becomes reckless and descends. The teaching which declares that men should be content in whatever condition they may severally find themselves, is subversive of advance, unphilosophical, and entitled to small respect. No man, unless dead to all aspiration by the embrutement of ignorance, can rest in absolute contentment. No community of men will be satisfied that its specialists, in whatever subdivision of employment, shall permit themselves to remain at an unprogressive standstill. It has been well said that if a horse knew as much as a man, it might not be so well for the rider, and if the rider knew as little as the horse, he would be an incompetent rider. So if a patient knows as much as his dentist, one would not like to be his dentist, and if the dentist knows as little as his patient, one would not like to be his patient. The specialist must be far in advance of those upon whom his specialty is to operate. Never before was this more requisite. Society was once content that the village barber should be the blood-letter, and the village blacksmith the tooth-puller. And the times of such ignorance men winked at and suffered accordingly. But these times, and these former shortcomings have forever passed away and never more will be tolerated. People of the present time are so increasedly educated that they would hold a man in derision, who, even in the agony of a torturing toothache, should run the risk of losing the wrong grinder by accepting the forceps of the wrong man. Now my application of these generalities to our profession is that we shall require, and rigidly demand, that the novices, the pupils who come to us to be disciplined in hand-work, shall have been previously so prepared by brainwork, that both our task and theirs shall be less perplexing; that the time they may continue with us shall be more profitably and more gainfully employed; that they shall begin with us with a well-disiplined intellect and a certain well-assorted and available stock of information; and not, as have too many heretofore, with a certain half-culture, half-knowledge, and half-development, which impedes our teaching and their learning. Let my advocacy not be misunderstood. I place a higher value upon the general discipline of the mind than upon the accumulation of special knowledge, for it is invariably true that whoever has acquired a good general education, and a ready working power of mind, finds the acquisition of any specialty a comparatively easy matter. In the construction of an edifice, each additional stone increases the bulk and adds to the height; but unless they be suitably joined together by a proper cement, and unless the foundation be deep and firm, the structure itself will not be strong. Our age is more dependent upon intellectual culture than any other. The supremacy of labor in the service of intellect over labor guided by mere habit and instinct, is established beyond dispute or argument. Every profession requires a general and a special preparation. Any special education demands a primary education as a preparation for and introduction to it. It is all essential that the foundation should be sufficient for the superstructure. In my own practice, I long ago resolved never to take as a student any one who had not received an education equal to that obtained by the course of study to be secured in our high schools. The graduate of a public high school has acquired an amount of actual culture nearly equal to that attained by those who have completed the second year in the course of study at the average college; and with this amount of mental discipline and knowledge, is well qualified to enter upon the study of the various branches of science taught in our medical and dental colleges, and will make correspondingly rapid progress. Without this, or some approximating amount of general culture, he finds himself hedged in and hampered about at every step in his course, unable readily to grasp or easily to retain the thoughts that are given him. In the study of anatomy and physiology, he is constantly having poured into his ears a succession of Latin and Greek terms, which he can neither pronounce, remember, nor comprehend, and he finds himself groping about continually in mental darkness. We look hopefully forward, therefore, to the day when all of our dental schools will have some high standard of general knowledge and scholarship as a requisite for matriculation, and not, as unfortunately is now the rule, allow any one possessing the required fee, regardless of their fitness for entering our profession, to make an attempt which must often end only in mediocrity of position or total failure. Give us better trained heads, and we will give the world better trained hands, and make American dentistry, now as a rule highest in repute, to be yet higher in the future.

To be born to be subjects of watchful care during growth, that growth being exposed to occasional interruption by disease, or what may be called disturbing and abnormal hindrances in reaching the climax of life; to decline, fade out, and to die, is the lot of every being and thing that exists. "The great globe itself, yea, and all which it inherits, shall dissolve and leave no track behind." This doom is the destiny affixed to all things and to all creatures by Him who created them, man being no exception. But with man, the power of reasoning from cause to effect, and from effect to cause, gives him the supreme advantage of being able, in some measurable degree, to impede the influences that cause decay, and, by postponing decay itself, to prolong the enjoyment of life. The ultimate fact of death itself we know cannot be averted. It is not only a fact, but it is a necessity. The earth, ere this, under the ordinary operation of increase, would long ago, but for death, have become so over-populated that, that very condition would have so intensified the demand for room, that the crowded masses of humanity would themselves have been compelled, in their push for room, to enter upon the work of thinning out, in which operation the docrine of the survival of the fittest would have found abundant support. And the only difference between *such* a condition and the condition which actually exists, is that the latter does the work of the former by a prolonged instead of a synchronous process; in a healthy instead of a baneful manner; and with the least possible disturbance of all the concomitants which attend the race. Disease and death, then, are necessities, and their certainty in any given case is only a question of time. Why, then, is there so much ado about the matter, and why should such effort be put forth to prevent this doom, a doom which no knowledge or skill can avert? Plainly because life is dear, and as a man of fourscore and ten years once said to me, "we all want to live a little longer." Now everybody knows that thousands die because of sheer ignorance of the ordinary laws which assure health; and thousands die, because, when disease attacks, their similar ignorance prevents the proper application of the right curative means, while all the time, in countless instances, pain and suffering are their normal conditions, because either ignorance or neglect overlook the means which not only the Creator, but simple common sense and common observation have clearly indicated as preventive means. It is conceded that great improvement has been attained, that the average of human suffering has, within a century, decreased, and that the average duration of human life has increased; and the acknowledged cause of each of these desirable conditions is the increase of general and special knowledge of both the physical and mental facts with which the Creator has surrounded and connected the wonderful apparatus called man, who since his creation has fallen into

> "A baser state than what was first assigned,
> Where best are lost and worst are left behind."

And all this goes to strengthen our general argument and to justify the demand we make, that yet greater knowledge in this direction shall be achieved. In fact we of this day shall be treacherous to the duty we owe to those who have preceded us, if we fail to supplement their work and to advance the cause for which they struggled and pleaded. If they achieved so much with the fewer facilities and the inferior means at their command, what may not we accomplish with our greater and better means? Now so intimately connected are all the several parts which make up that extraordinary complexity called the human frame, that the ill-health of one part is sure to act unfavorably upon another. St. Paul put the matter with philosophical accuracy when he said, "whether one member suffer, all the members suffer with it." And Shakespeare rightly declares that "one twinge of the tooth-ache would make the mighty Cæsar forget that he was Emperor of the world," proving conclusively the intimate connection between the physical and mental in man—between the teeth that grind his food and the memory that holds his facts. Burns follows it all up by venting his

"Curse upon the venomed stang,
That knaws my tortured gums alang,
And thro' my lugs gies many a twang
Wie knawing vengeance."

Infinite wisdom and infinite power produced, as their last and best work, the very image and glory of God. "Noble in reason, infinite in faculties, in form and motion express and admirable; in action like an angel, in apprehension like a God." And yet this marvelous work, by reason of the added causes of ignorance, neglect, and the lack of apt appliance of what experience through the long ages ought to have taught to the average observation, is the victim of disease induced by trifling causes, and of death by premature decay. A breath of air may be the storm of its destiny, or a dewdrop the flood that destroys it, and "when his greatness is a ripening, then a frost nips his root and he falls." Now conceding, as we must, that death is a necessary means of thinning out, an indispensable preventor of overcrowding, it has been well demonstrated that neither death nor thinning out demands encouragement; that human progress and human happiness may each and both of them be increased by the application of such means as experience has indicated and science deduced and assured, we come back to our proposition, that education, in the general and in the detail, is the agency by which we must control and avert any unnecessary rapidity in the operation of the inevitable law of decay. And in its application to our own profession, I am daily confirmed in the opinion that both duty and good policy, and even common honesty of dealing in our specialty demand, and will (as it ought to do) insist upon advanced culture in every man who enters thereupon. Now to build up as a science, what has hitherto but ranked as an art, and in its early adventurings as a rather rough, clumsy, and dreaded art, we who have witnessed its growth in applied experience, in improved instrumentalities, in skill of application, in comfort of means used, in the banishment of fear and pain, must insist upon further protection of the suffering by increased preparation upon the part of our novitiates in the profession. And a most important and surely a most welcome means of achieving this increased preparation would be an antecedent education in the general science of medicine, that itself to be supplemented by a thorough knowledge of the organic framework of the body and of human physiology. In the general medical profession, the last half century has witnessed an important and interesting change, particularly in centres of denser populations. This change is in accordance with the methods of general business operations,—that of subdivision and specializing. Merchants of large experience have learned that devotion to a single line of business has enabled them to do better for their customers and better for themselves. He whose whole mind and time are bent upon the effort to perfect a single class of merchandize, and who, as a normal certainty, secures such perfection, establishes a reputation that infallibly allures, his general knowledge as a merchant being his most efficient auxiliary. It is equally true that the medical man who selects some department of the great field of Therapeutics as a specialty, deriving similar aid in that specialty from his preliminary knowledge of general medicine, will be vastly more sure of success, and success in the vocation of medicine, in any of its departments, is but another name for beneficence to man. Let us suppose that a man thus fore-trained and fore-armed, intends to enter the field of dentistry. He first places himself for the general mechanics of his intended avocation, under the instruction of an experienced adept. Now it is at once apparent that the master will have less work to do in the matter of instructing, and the pupil less to do in the matter of learning, even in manual work, than if he came in the raw and immature. For if he so come, he would have to learn not only the mere mechanics of the art, the manual of its ways and means, but the theory upon which all these appliances depend, and their adaptedness to physiological exigency. Now inverting this reasoning we say that the justifiable demands of the profession claim what has too long been ignored, that the candidate shall be better prepared for his apprenticeship, and therefore a more teachable pupil for his master; and therefore more easily receptive of education in our art; and therefore more readily and thoroughly made an artist, expert and ready in resource; and therefore more acceptable to the community, more trusted and more safe; and therefore more valued and honored in the profession, inasmuch as all his influences and doings tend to its elevation.

There is an ancient Roman legend, that after one of the many battles of the republic, a review of the victorious troops was held by the Consul. As the leading column, composed of scarred veterans, with bronzed faces and grizzly beards,—men who had borne the brunt of battle upon many a hotly contested field,—as they passed by beneath the standard to which, as veterans, they were entitled, they shouted, "We *have* been brave; we *have* been brave." "Yes," replied the Consul, "you have indeed been brave, the very bravest, and you made Rome what she is, the mistress of the world, its pride and its glory. But when I reflect that you are soon to pass away, I tremble lest her glory and her safety be lost." Next marched by a firm-paced column of the troops, younger in years and in service, but stronger in physique and of courageous heart, waiving aloft their emblems of victory, and shouting with full voice, "We *are* brave, we *are* brave." "Yes, yes," responded the Consul, "you are also brave, and Rome honors your bravery. But when I consider that, ere long, you must succeed to the places of the veterans who have preceded you, and that you, like them, will ere long be lost to Rome, I fear for the safety and permanence of the republic." But who are these that are moving on in firmly-serried ranks, with elastic tread, with cheerful and confident bearing? Ah! these are the gallant youths of Rome, who will, as did their fathers, see to it that the republic receives no harm. Hark to their shout: "We *will* be brave, we *will* be brave." "Thanks to the Gods," the aged Consul exclaimed, as tears gushed from his eyes, "if the young are to *be* brave, then indeed is the perpetuity and glory of Rome secured." I see before me many veterans in our profession who have done what their times and their means permitted, in raising it from mere manual work to a profession; from obscurity to eminence. We accord them highest praise for highest success in pioneering. So, too, I see many a successor to these pioneers, who, earnestly following up and supplementing their good work, still further advanced the banners of our tribe, and planting this profession upon an abiding foundation. To them be given highest praise for the success and the good they have achieved. And again I see in gathering numbers a denser array, with eager step and under better auspices, crowding into our ranks. Their rallying cry should be, "we *will* be brave, we *will* be true, and bravely and truly maintain the honor and dignity of the profession upon which we are entering." This, young man, is your chief duty, and in the performance of that duty those who have preceded you, and who have bequeathed to you the work of still further advancing the profession, will hold you to strictest responsibility. See that you fail not in the duty you owe to our predecessors and your own.

